# Stress-Induced Chronic Visceral Pain of Gastrointestinal Origin

**DOI:** 10.3389/fnsys.2017.00086

**Published:** 2017-11-22

**Authors:** Beverley Greenwood-Van Meerveld, Anthony C. Johnson

**Affiliations:** ^1^Oklahoma Center for Neuroscience, University of Oklahoma Health Science Center, Oklahoma City, OK, United States; ^2^Department of Physiology, University of Oklahoma Health Science Center, Oklahoma City, OK, United States; ^3^VA Medical Center, Oklahoma City, OK, United States

**Keywords:** stress, pain, colon, animal model, gastrointestinal tract, irritable bowel syndrome, brain, early life

## Abstract

Visceral pain is generally poorly localized and characterized by hypersensitivity to a stimulus such as organ distension. In concert with chronic visceral pain, there is a high comorbidity with stress-related psychiatric disorders including anxiety and depression. The mechanisms linking visceral pain with these overlapping comorbidities remain to be elucidated. Evidence suggests that long term stress facilitates pain perception and sensitizes pain pathways, leading to a feed-forward cycle promoting chronic visceral pain disorders such as irritable bowel syndrome (IBS). Early life stress (ELS) is a risk-factor for the development of IBS, however the mechanisms responsible for the persistent effects of ELS on visceral perception in adulthood remain incompletely understood. In rodent models, stress in adult animals induced by restraint and water avoidance has been employed to investigate the mechanisms of stress-induce pain. ELS models such as maternal separation, limited nesting, or odor-shock conditioning, which attempt to model early childhood experiences such as neglect, poverty, or an abusive caregiver, can produce chronic, sexually dimorphic increases in visceral sensitivity in adulthood. Chronic visceral pain is a classic example of gene × environment interaction which results from maladaptive changes in neuronal circuitry leading to neuroplasticity and aberrant neuronal activity-induced signaling. One potential mechanism underlying the persistent effects of stress on visceral sensitivity could be epigenetic modulation of gene expression. While there are relatively few studies examining epigenetically mediated mechanisms involved in visceral nociception, stress-induced visceral pain has been linked to alterations in DNA methylation and histone acetylation patterns within the brain, leading to increased expression of pro-nociceptive neurotransmitters. This review will discuss the potential neuronal pathways and mechanisms responsible for stress-induced exacerbation of chronic visceral pain. Additionally, we will review the importance of specific experimental models of adult stress and ELS in enhancing our understanding of the basic molecular mechanisms of pain processing.

## Introduction

Chronic pain is defined as pain lasting longer than 3 months after the resolution or in the absence of an injury. Chronic visceral pain describes persistent pain emanating from the thoracic, pelvic, or abdominal organs that is poorly localized with regard to the specific organ affected. Here we will briefly review visceral pain pathways and their modulation by (i) stress in adulthood and (ii) following exposure to neonatal stress. We will provide an evidenced-based argument for the use of specific experimental models to advance the understanding of stress-induced chronic pain. We will explain the unique aspects of these models that allows for a carefully crafted investigation of the female vulnerability to stress-induced chronic visceral pain. Although the science of the epigenetics of human pain management is in its early stages with relatively few studies that have examined epigenetically mediated mechanisms involved in nociception in human subjects, a key aspect of the review will be to highlight the latest insights into epigenetic processes, including DNA methylation, histone modifications and microRNAs, and describe their involvement in the pathophysiology of chronic visceral pain.

## Visceral pain pathways

Pain originating from the gastrointestinal (GI) system ascends to the brain via the same tri-neuronal pathways that convey noxious somatic stimuli. In the GI tract, nociceptive neurons, with cell bodies in the dorsal root ganglion (DRG), have free nerve endings that generally contain multiple receptor types that respond to various modalities, such as pH, stretch, temperature, or the addition of specific chemicals such as chronic stress mediators (Million et al., 2006; Ochoa-Cortes et al., 2014; Vanner et al., 2016). Some of the receptors are cation channels, which can directly depolarize the nociceptor upon activation, while other receptors activate second messenger systems to change neuronal excitability by changing expression of, or modifying the function of, other cation channels. Nociceptors innervate all layers of the GI tract; nerve endings in the mucosa can be activated by luminal contents (digestive materials, bacteria, or bacterial metabolic products), or by signaling from enterochromaffin cells; nerve endings in the submucosal or the myenteric plexus are typically activated by local release of neurotransmitters and neuromodulators from intrinsic nerves or by resident immune cells; nerve endings within the muscle layers or blood vessels are typically activated by noxious stretch (Brookes et al., 2013; Barbara et al., 2016; Vanner et al., 2016). A small proportion of nociceptive neurons have dichotomous afferents that innervate both GI and adjacent organs, such as the bladder or overlying skin dermatomes (Schwartz and Gebhart, 2014). Once initiated within the periphery, the noxious signal is transmitted to the dorsal horn of the spinal cord where the first synapse occurs. Typically, the peripheral nociceptive neuron synapses on a cell body of a projection neuron within the superficial lamina of the dorsal horn of the spinal cord, the substantia gelatinosa or the nucleus proprius; however, unlike noxious signals arising from somatic structures synapsing at a specific spinal level, visceral afferents may synapse at multiple spinal levels, leading to diffuse localization of the noxious signal (Schwartz and Gebhart, 2014). Within the dorsal horn, the ascending pain signal can be modulated by local inhibitory interneurons and by descending projections from the brain stem (the periaqueductal gray, Raphe, or medulla) (Heinricher et al., 2009; Kuner, 2010; Denk et al., 2014). The projection neuron then sends a process to the contralateral side of the spinal cord to ascend to the brain in the anteriolateral columns, although some visceral afferent signals can also ascend in the ipsilateral dorsal columns (Palecek, 2004). The ascending fibers of the second order neurons are organized into the spinothalamic, the spinoparabrachial, and the spinoreticular tracts, depending on where the cell body of the third-order neuron is located (Almeida et al., 2004). The final primary synapse occurs at cell bodies within the brain. For the spinothalamic tract, the 3rd order neuron is within the thalamus, which acts as the primary hub for the central pain matrix (Morton et al., 2016). The thalamus is somatotopically organized such that noxious signals from the spinal cord are sent to specific regions of the primary somatosensory cortex for the localization of the signal. In contrast, the cortical localization for visceral pain is typically less precise since the ascending signal often innervates the spinal cord at multiple levels and pain signals from visceral and somatic sources may be transmitted by the same 2nd order spinal neuron (viscerosomatic convergence). Within the central pain matrix, the thalamus signals to brain regions that process the emotional component of the pain signal, such as the amygdala, insula, anterior cingulate cortex, hippocampus, and nucleus accumbens (Wilder-Smith, 2011; Bushnell et al., 2013). In healthy individuals, activation of the central pain matrix provides the appropriate behavioral responses (unpleasant emotion, guarding, and/or immobilization of the affected site) to promote recovery and to learn avoidance to prevent future injury (Navratilova and Porreca, 2014). Descending antinociceptive brainstem pathways are also activated by the central pain matrix to decrease noxious signaling at the dorsal horn of the spinal cord by changing the excitability of the 2nd order neuron within the spinal cord (Heinricher et al., 2009; Denk et al., 2014).

## Mechanisms responsible for chronic visceral pain

Sensitization of the primary (peripheral afferent), secondary (spinal), or tertiary (brainstem/thalamic) neuron can promote chronic pain signaling (Fornasari, 2012). For visceral pain, peripheral sensitization can occur in response to tissue injury or due to release of inflammatory mediators (chemokines, corticotropin-releasing hormone, cytokines, histamine, proteases, prostaglandins, serotonin) in response to injury or infection (Arroyo-Novoa et al., 2009; Widgerow and Kalaria, 2012). Furthermore, in response to the peripheral stimuli, the afferent fibers can release neuromodulators (calcitonin gene related peptide, nitric oxide, substance P) that act as paracrine agents to further stimulate nerve activity. Prolonged exposure to these mediators leads to activation of second messenger signaling cascades that can alter phosphorylation and/or expression receptors (particularly cation channels), promoting persistent changes in the electrical properties of the neuron such as lowering action potential threshold or increasing the number of action potentials upon reaching threshold (Woolf and Salter, 2000). Since neuronal sensitization is induced by activation of G-protein coupled receptors that respond to algesic chemicals, and they signal through common second messenger systems, pharmaceuticals targeting these systems could represent a novel approach to treat chronic pain conditions (Reichling and Levine, 2009). Alternatively, the cellular excitability is maintained by ion channels with kinetic properties or expression patterns altered by the second messenger phosphorylation systems, which also represents valid targets for new therapies for chronic pain (Schaible et al., 2011; Stemkowski and Smith, 2012).

Following sensitization of primary nociceptive afferents, the enhanced neuronal excitability increases neurotransmitter and neuromodulator release within the dorsal horn of the spinal cord. Typically the nociceptors use glutamatergic signaling, to activate ionotropic α-amino-3-hydroxy-5-methyl-4-isoxazolepropionic acid (AMPA) and N-methyl-d-aspartate (NMDA) receptors on the target neuron, with co-release of neuromodulators, such as substance P, to activate similar second-messenger systems to modulate the function of the second order neuron (Woolf and Salter, 2000). The primary function of the second messenger signaling to is to change the expression of AMPA and NMDA receptors, in particular by recruiting a calcium permeable AMPA receptor variant to the plasma membrane to increase the overall excitability of the neuron (Tao, 2012). Thus, even if the peripheral nociceptive afferent is able to reverse the sensitization once the acute injury has healed, the second order neuron will still be able to reach action potential thresholds with less neurotransmitter release from the primary afferent due to the increased calcium permeability. A second, concurrent mechanism to promote dorsal horn sensitization occurs due to disinhibition of inhibitory interneurons, either due directly from signaling from the primary afferent affecting receptors on the inhibitory interneuron, or indirectly by decreasing the endogenous descending noxious inhibition from the brainstem (Zeilhofer et al., 2012; Braz et al., 2014). Overall, increased peripheral excitability with decreased inhibitory tone leads to remodeling and persistent excitation of the second order neuron, which promotes chronic pain.

Finally, sensitization of the central pain matrix can directly promote and maintain chronic pain. The central pain matrix is composed of the limbic and cortical brain areas that respond to the emotional and physical components of pain. Increased afferent nociceptive neurotransmission due to peripheral or spinal sensitization can invoke similar central remodeling to cause persist pain (Jaggi and Singh, 2011). Initially, the 3rd order neurons within the thalamus or brainstem receive the enhanced activity from the spinal cord, causing additional signaling to the other cortical and limbic regions of the pain matrix. The integration nuclei, such as the amygdala, hippocampus, insula, or cingulate, are subsequently sensitized in response to the increased afferent stimulation, which can change activation thresholds, change previously innocuous stimuli to be perceived as noxious, and cause the negative emotional responses to chronic pain (Staud, 2012). An additional consequence of the central remodeling of the pain matrix is the loss of descending inhibition of the ascending noxious signals, which has been demonstrated in brain imaging studies of patients with chronic pain conditions, including visceral pain disorders (Ossipov et al., 2000; Heinricher et al., 2009; Wilder-Smith, 2011; Saab, 2012).

## Stress modulation of central pathways in chronic visceral pain

While the previous description was presented as a “bottom-up” model of sensitization leading to chronic visceral pain, direct sensitization of the central pain matrix can drive a “top-down” mechanism wherein stress and negative emotions can promote enhanced perception of nociception in the absence of overt peripheral injury (Scarinci et al., 1994; Lampe et al., 2003; Maizels et al., 2012; Racine et al., 2012). The body's response to stress is composed of two parallel systems: the quick “flight or fight” of the sympathomedullary axis and the slower hypothalamic-pituitary-adrenal (HPA) axis. The sympathetic response to acute stress mobilizes epinephrine and norepinephrine to change blood-flow away from the skin and GI tract toward the muscles along with providing a burst of energy and a dampening of pain perception to allow the individual to run or fight for survival. The neuroendocrine response mediated by the HPA axis causes release of cortisol in humans or corticosterone in rodents (CORT) to mobilize glucose reserves to restore homeostasis after an acute stressor, or to cause long-term changes in metabolic function and neuronal sensitivity following chronic stressors. Typically, the sympathetic response will habituate to repeated stressors, whereas the HPA response may or may not habituate depending on the type, duration, and variability of the stressor. As implied by the name, the HPA axis is initiated when paraventricular nucleus of the hypothalamus secretes the 41-amino acid peptide corticotropin-releasing hormone (CRH) into the hypophyseal portal circulation in response to a stressor. After binding to corticotrophs in the anterior pituitary, CRH causes the release of the 39-amino acid peptide adrenocorticotropic hormone into the systemic circulation after being cleaved from its 241-amino acid precursor protein, proopiomelanocortin. After binding in the adrenal cortex, adrenocorticotropic hormone induces *de novo* synthesis of CORT from a cholesterol-derived steroid precursor, which then enters systemic circulation bound to a carrier protein (cortisol binding globulin). In addition to its metabolic functions, CORT binding to its high affinity mineralocorticoid receptor (MR) and low affinity glucocorticoid receptor (GR) within brain regions such as the hippocampus, the paraventricular nucleus of the hypothalamus, and some cortical regions induces negative feedback to terminate the response of the HPA axis, while binding at the amygdala opposes the feedback inhibition by increasing CRH expression and facilitation of the stress axis (Sapolsky et al., 1983; Reul and de Kloet, 1985; Herman and Cullinan, 1997; Schulkin et al., 1998; Shepard et al., 2000). In particular, the central nucleus of the amygdala (CeA) integrates viscerosensory signaling with neuroendocrine and autonomic responses to stressors, and is primed to influence both stress and pain signaling though expression of MR, GR, and CRH (Myers and Greenwood-Van Meerveld, 2010a, 2012; Johnson and Greenwood-Van Meerveld, 2015; Johnson et al., 2015). Additionally, following chronic stress exposure, evidence points to neuronal remodeling that is region specific. For example, in a stress-inhibitory region, such as the hippocampus, dendritic structure is simplified (less synaptic connections, weakened circuit) whereas in the stress-facilitatory amygdala dendrites becomes more complex in structure (more synaptic connections, strengthened circuit) (Woolley et al., 1990; Vyas et al., 2002; Mitra and Sapolsky, 2008; Radley et al., 2013). The likely net effect of this neuronal remodeling following chronic stressors is the exacerbation of pain perception and the promotion of chronic pain symptomatology due to the loss of anti-nociceptive and anti-stress signaling within the central pain matrix combined with facilitation of nociceptive and stress-responsive signaling. These remodeled pain circuits also impinge on the function of key brainstem regions that modulate descending pain inhibition. The periaqueductal gray and rostral ventral medulla form an integrative circuit that modules ascending pain signals with function influenced by inhibitory connections from cortical brain regions and facilitatory connections from the amygdala (da Costa Gomez and Behbehani, 1995; Price, 1999). A direct integration of the sympathomedullary and HPA axis is achieved by CRH-ergic connections from the amygdala to the locus coeruleus and norepinephrine-ergic connections from the locus coeruleus to the amygdala (Reyes et al., 2011). Clinically, the effect of chronic stress on visceral pain is best illustrated by the high co-morbidity of anxiety, depression, and other psychiatric disorders with functional pain disorders, such as irritable bowel syndrome (IBS) (Drossman et al., 2011; Hooten, 2016). Because there are multifactorial mechanisms that can induce chronic visceral pain, further research is necessary to identify specific mechanisms underlying the development of chronic stress-induced visceral pain.

## Neurotransmitters in stress pathways that modulate visceral nociception

**Figure 1:**
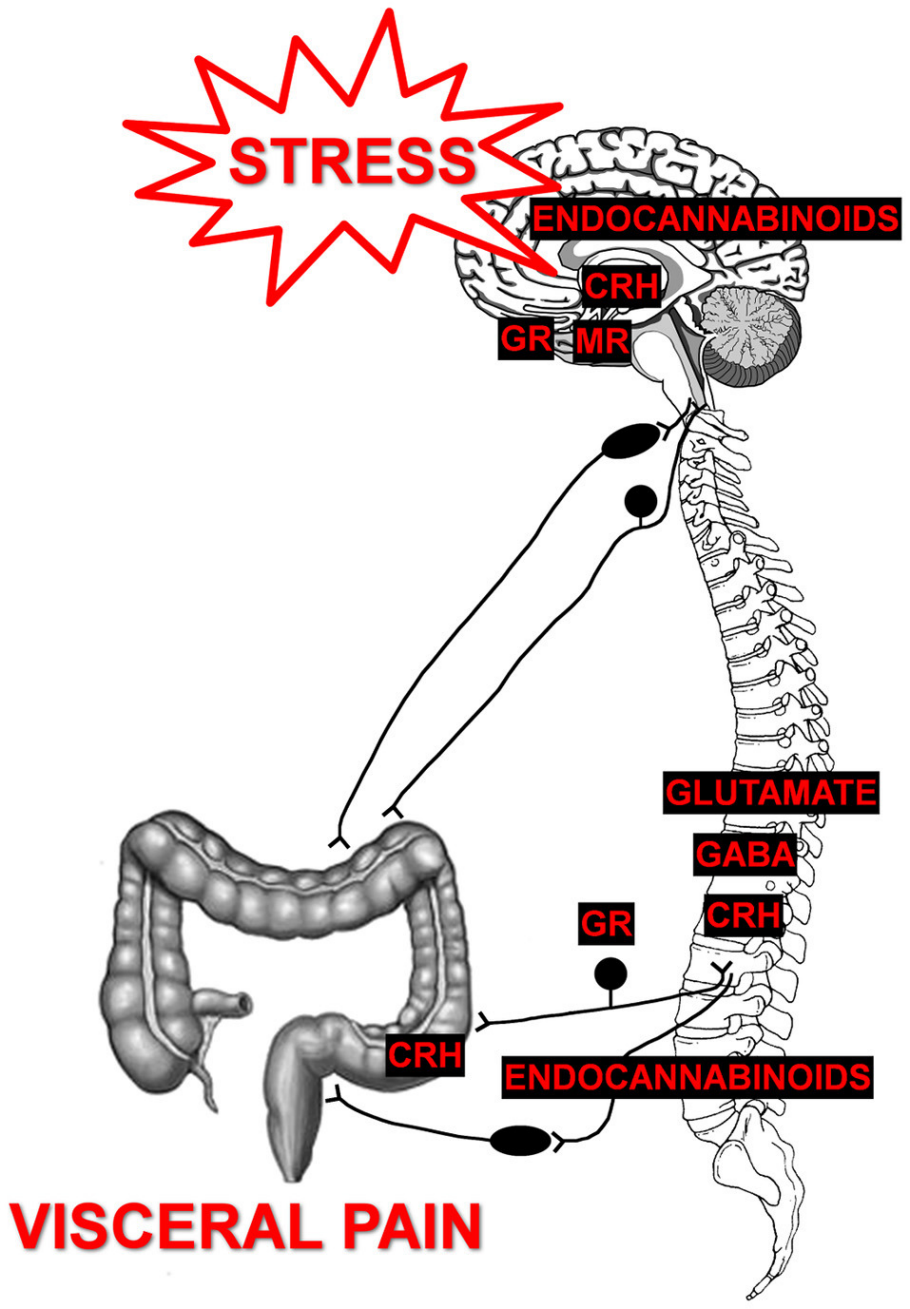
Mediators of chronic stress-induced visceral pain. Prolonged exposure to stressor can cause central dysregulation of the hypothalamic-pituitary-adrenal (HPA) axis by changing the expression of glucocorticoid receptors (GR) and mineralocorticoid receptors (MR) in limbic brain areas, such as the amygdala. Such changes lead to increased expression of corticotropin-releasing hormone (CRH), which facilitates further activation of the HPA axis and neuronal sensitization of the central pain matrix. Stress also disrupts endocannabinoid signaling that participates in fast-feedback inhibition of the HPA axis to modulate neuronal sensitivity within with the central pain matrix. Preclinical studies in visceral and neuropathic pain models have demonstrated roles for CRH to modulate spinal sensitization as well as GABA-ergic and glutamatergic signaling to modulate spinal sensitization to promote chronic pain. Within the dorsal root ganglia, roles for endocannabinoid signaling modulated by the GR have been demonstrated models of stress-induced pain. Additionally, local release of CRH within the enteric nervous system can modify sensitivity of extrinsic primary afferents to distension. Thus, multiple neurotransmitters, neuromodulators, and/or stress-responsive receptors are activated by chronic stressor leading to the development of chronic visceral pain.

Multiple studies using preclinical models of experimentally induced stress have identified a plethora of neurotransmitters and neuromodulators capable of promoting stress-induced visceral hypersensitivity. An exhaustive description of every target is beyond the scope of the current review, but many recent publications have highlighted some of the various modulators (Mora et al., 2012; Asan et al., 2013; Reichling et al., 2013; Timmermans et al., 2013; Grace et al., 2014; Greenwood-Van Meerveld et al., 2015). Here we aim to focus on specific targets within both the stress and pain circuits that play a key role in the development of visceral hypersensitivity in chronic visceral pain models (Figure 1).

### Role of corticosteroids in stress-induced visceral hypersensitivity

GR and MR are in the 3-ketosteroid nuclear receptor superfamily that also includes androgen and progesterone receptors (Lu et al., 2006; Alexander et al., 2015a). Originally, GR and MR were characterized as cytoplasmic transcription factors responsible for changing cellular processes over hours, days, or weeks due to changes in expression of target genes (de Kloet et al., 2005). More recently, there is compelling evidence for membrane bound versions of both GR and MR leading to changes in function within minutes of binding, including participating in regulation of feedback inhibition of the HPA axis (Di et al., 2003; Haller et al., 2008; Evanson et al., 2010; Prager et al., 2010; Hammes and Levin, 2011). Within the brain, MR expression is restricted to key nuclei such as the hippocampus and amygdala. In contrast, GR is expressed throughout the brain, and both the cytoplasmic and membrane versions are typically present to balance the fast and slow effects of either receptor to produce the correct response to an acute stressor (Johnson et al., 2005; Karst et al., 2005; Lu et al., 2006; Evanson et al., 2010; Prager et al., 2010). Following neuronal remodeling by chronic stress, the balance between the effects of the receptors is disrupted, promoting chronic stress-induced pain due to altered signaling within the central pain matrix (Johnson and Greenwood Van-Meerveld, 2012). There are many studies demonstrating that manipulating GR or MR signaling with selective antagonists or via knockdown of receptor expression can modulate neuropathic pain circuits, particularly those with a spinal site of action (Wang et al., 2004; Takasaki et al., 2005; Gu et al., 2007; Dina et al., 2008; Dong et al., 2012).

Multiple studies suggest that stress-induced changes in GR and/or MR expression within the nervous system (DRG, spinal cord, or brain) can directly affect colonic sensitivity, suggesting that dysregulation of these receptors participates chronic visceral pain. In support, we have shown that exposing GR and MR receptors in the CeA, via stereotaxic application, either to the non-selective agonist CORT, to the selective GR agonist dexamethasone, or to the selective MR agonist aldosterone, induces colonic hypersensitivity with inhibition of the effects through co-application of selective antagonists (Myers and Greenwood-Van Meerveld, 2007, 2010a; Myers et al., 2007). Using the stereotaxic implantation model, we found that persistent visceral hypersensitivity was associated with a long-term decrease in GR expression within the CeA (Tran and Greenwood-Van Meerveld, 2012). Building upon these observations, we were the first to demonstrate that a central epigenetic mechanism within the CeA involving changes in histone acetylation was responsible for the long-term decrease in amygdala GR expression and persistent colonic hypersensitivity (Tran et al., 2015). In a similar fashion, repeated exposure of a rat to water avoidance stress (WAS) induced changes in methylation of the GR promoter within the CeA leading to decreased GR expression and colonic hypersensitivity through a mechanism involving an increase in CRH (Tran et al., 2013). The WAS-induced colonic hypersensitivity could also be inhibited by stereotaxic application of selective GR or MR antagonist to the CeA (Myers and Greenwood-Van Meerveld, 2012), or following systemic administration of a GR antagonist (Hong et al., 2011). The role of GR and MR receptors in the CeA for the modulation of colonic sensitivity was demonstrated by mimicking the stress-induced decrease in expression of the receptors through the use of selective antisense oligodeoxynucleotides to knockdown expression of either receptor in the CeA, which was sufficient to induce colonic hyperalgesia in stress-naïve rats (Johnson and Greenwood-Van Meerveld, 2015). Further evidence was reported in female rats exposed to early-life stress (ELS) with colonic hypersensitivity in adulthood that displayed an increase, rather than a decrease, in GR expression within the CeA; however, the ELS-induced colonic hypersensitivity was exacerbated following GR mRNA antisense oligodeoxynucleotides administration into the CeA, suggesting additional neurotransmitter systems underlying ELS-induced colonic hypersensitivity (Prusator and Greenwood-Van Meerveld, 2017). In a two-hit model of colonic hypersensitivity induced by both neonatal and adult noxious colonic distension, reduced hippocampal GR expression was associated with colonic hypersensitivity (Zhang et al., 2016b). Further evidence for a central regulation of ELS-induced colonic hypersensitivity was reported in a model of maternal separation in which colonic hypersensitivity in adult male rats was inhibited via an acute microinjection of either a GR or MR antagonist into the right CeA (Zhou X. P. et al., 2016). There is also experimental evidence for a role for steroid receptors within the periphery in the development of stress-induced colonic hypersensitivity (Hong et al., 2011). In response to repetitive exposure to a water avoidance stressor, male rats exhibited a decrease in GR expression in L6-S2 DRG. Subsequent experiments from the same investigators found that the decrease in GR expression was due to increased methylation of the GR promoter pointing to an epigenetic mechanism at the level of the spinal cord in the induction of chronic visceral hypersensitivity (Hong et al., 2015).

### Role of corticotropin-releasing hormone (CRH) in stress-induced visceral hypersensitivity

CRH is released from the paraventricular nucleus of the hypothalamus to initiate the HPA axis in response to stress, and is also highly expressed within the CeA (Gallagher et al., 2008). CRH binds to its high-affinity CRH type 1 receptor (CRH_1_) and its lower affinity CRH type 2 receptor (CRH_2_), both of which are G-protein coupled receptors (Alexander et al., 2015b). While activation of both receptors increases intracellular cAMP, they produce opposing effects on behavior; CRH_1_ activates the stress response and enhances nociception, while CRH_2_ inhibits the stress response and decreases nociception in rodents (Ji and Neugebauer, 2007, 2008; Yarushkina et al., 2009; Tran et al., 2014). Clinical studies have found that CRH within the cerebrospinal fluid was positively correlated with pain perception in patients with chronic pain (McLean et al., 2006). A non-selective CRH antagonist decreased pain induced by colonic distension, while intravenous CRH induced esophageal hypersensitivity to distension in healthy volunteers, with no changes in perception of other noxious stimuli (Sagami et al., 2004; Broers et al., 2017). Intravenous CRH also increased abdominal pain and amygdala blood flow in both healthy volunteers and IBS patients (Tanaka et al., 2016). In adult rodent models of stress, there is evidence for modulation of colonic hypersensitivity by both central and peripheral CRH receptors. We have shown that CRH expression in the CeA acting via CRH_1_ receptors mediates colonic hypersensitivity in adult female rats following neonatal exposure to ELS as well as persistent colonic hypersensitivity induced by adulthood stress in male rats (Johnson et al., 2015; Prusator and Greenwood-Van Meerveld, 2017). Further supporting evidence for a role of amygdala CRH in stress-induced visceral sensitivity demonstrated that intra-CeA CRH administration increased colonic sensitivity via CRH_1_ receptor activation in stress-naïve male rats (Su et al., 2015). Peripheral administration of a CRH_1_ antagonists dose-dependently reduced stress-induced colonic sensitivity and decreased stress-induced fecal pellet output (Million et al., 2013; Taguchi et al., 2017). Similarly, a peripherally restricted CRH_1_ agonist induced colonic hypersensitivity in stress-naïve adult male rats while a CRH_2_ antagonist was also able to inhibit stress-induced colonic hypersensitivity (Larauche et al., 2009; Nozu et al., 2014; Mulak et al., 2015). There is strong supportive experimental evidence for an important role for CRH in chronic visceral hypersensitivity following exposure to early life adversity. In models of early life stress, male mice exposed to maternal separation or neonatal noxious colonic distension developed colonic hypersensitivity in adulthood that was associated with increased CRH expression within the paraventricular nucleus of the hypothalamus (Zhang et al., 2016a; Tang et al., 2017). In male rats that underwent neonatal noxious colonic distension, adult colonic hypersensitivity was associated with increased CRH expression in the colon, spinal cord, and brain (Liu H. R. et al., 2015). In another experimental model of ELS in which male pups receive repetitive enemas of dilute acetic acid to induce visceral hypersensitivity in adulthood, intracerebroventricular administration of a non-selective CRH receptor antagonist significantly inhibited adult colonic hypersensitivity (Jia et al., 2013). Thus, dysregulation of CRH signaling throughout the brain-gut axis can induce colonic hypersensitivity following neonatal and/or adult stressors, emphasizing the importance of this system in the maintenance of chronic visceral pain.

### Role of endocannabinoids in stress-induced visceral hypersensitivity

The endogenous endocannabinoids, anandamide and 2-arachidonoylglycerol (2-AG), bind to two G-protein coupled receptors, cannabinoid receptor type 1 (CB_1_) and type 2 (CB_2_) (Pertwee et al., 2010; Alexander et al., 2015b). CB_1_ and CB_2_ are differentially expressed, with CB_1_ being the major central receptor that can modulate stress and pain perception, while CB_2_ is the major peripheral receptor with an unclear role in stress and pain (Gorzalka et al., 2008; Butler and Finn, 2009; Hill and McEwen, 2010; Luongo et al., 2014). In addition to the receptors, drugs that inhibit the major endocannabinoid degrading enzymes, fatty acid amide hydrolase (FAAH) for anandamide and monoacylglycerol lipase (MAGL) for 2-AG, as well as fatty acid binding proteins that carry anandamide and 2-AG, are also therapeutic targets for stress-induced pain (Patel et al., 2017; Woodhams et al., 2017). Evidence for a role of endocannabinoids in visceral hypersensitivity emanates from studies in experimental models. Recently, a novel FAAH, MAGL, or a combined FAAH/MAGL antagonist were investigated in visceral pain models. Only compounds with either FAAH antagonism activity or a dual CB_1_/CB_2_ agonist provided a robust inhibition of colonic hypersensitivity (Sakin et al., 2015). A selective FAAH antagonist completely inhibited acetic acid-induced writhes and significantly inhibited colonic mustard oil-induced writhes in male mice, through a CB_1_ sensitive mechanism, with no drug effect in FAAH knockout mice (Fichna et al., 2014). A selective MAGL antagonist dose-dependently inhibited phenylbenzoquinone-induced writhes in mice, which was reversed by a CB_1_ antagonist with no effect of a CB_2_ antagonist; however, the same doses that were effective at reducing visceral pain disrupted short-term memory (Griebel et al., 2015). MAGL knockout mice demonstrated significantly increased writhes in response to acetic acid, which was inhibited by a CB_1_ antagonist, while repeated dosing of a MAGL inhibitor was able to exacerbate the writhing response in wildtype mice (Petrenko et al., 2014). Based on co-localization within the myenteric plexus, a novel compound acting as both a FAAH and TRPV1 antagonist significantly inhibited protease activated receptor-2-induced colonic hypersensitivity in male mice, although the effect was not reversed by a selective CB_1_ antagonist (Bashashati et al., 2017). In rats exposed to repeated stress induced by water avoidance, colonic hypersensitivity was associated with a region specific loss of CB_1_ expression and increase in TRPV1 expression in C-fibers of L6-S1 DRGs, along with a local increase in 2-AG content and a decrease in FAAH expression within the DRG (Zheng et al., 2015). Intrathecal delivery of small interfering RNA (siRNA) to knockdown DNA methyltransferase 1 prevented the decrease in CB_1_ expression, while knockdown of the histone acetyltransferase EP300 inhibited the increase in TRPV1 expression, and either siRNA was able to inhibit WAS-induced colonic hypersensitivity (Hong et al., 2015). In female mice, exposure to water avoidance stress increased CB_2_ mRNA expression within the colon (Aguilera et al., 2013). Taken together, these preclinical findings suggest that modulation of the endocannabinoid system is capable of affecting visceral sensitivity and thus may play a role in the chronic stress-induced visceral pain. There is some very limited clinical evidence supporting abnormalities in the endocannabinoid system in chronic functional GI pain disorders. An interesting pilot study in 12 patients with pain-associated functional dyspepsia showed increased central availability of CB_1_ receptors, as measured via positron emission tomography scanning, compared to healthy volunteers (Ly et al., 2015). Similarly, in another pilot study of 14 IBS patients compared to seven healthy volunteers, there was a significant decrease in FAAH mRNA from colonic biopsies in the IBS patients suggesting that the endocannabinoid system may play a role in the pathophysiology of IBS (Fichna et al., 2013). Due to the preliminary nature of these clinical studies, additional studies are required in larger cohorts of patients to fully define the role of the endocannabinoid system in functional pain disorders.

### Role of gamma-amino butyric acid (GABA) in stress-induced visceral hypersensitivity

Receptors for GABA are divided into two functional classes: GABA type A (GABA_A_) receptors are ionotropic chloride channels, while GABA type B (GABA_B_) receptors are metabotropic G-protein coupled receptors (Bowery et al., 2002; Olsen and Sieghart, 2008; Alexander et al., 2015b,d). Both receptors are expressed in pain and stress-responsive neural circuits with effects on those systems demonstrated through the use of selective agonists and antagonists (Enna and Bowery, 2004; Goudet et al., 2009; Munro et al., 2013; Gunn et al., 2014). In rodent models, oral administration of a *Bifidobacterium* sp. genetically engineered to produce GABA resulted in the normalization of electrophysiological properties of colonic nociceptors in a model of chronic colonic hypersensitivity (Pokusaeva et al., 2017). Spinal administration of a selective GABA_Aα−2_ agonist inhibited colonic sensitivity to distension in female rats with a normosensitive colon (Kannampalli et al., 2017a). A similar finding was demonstrated with intrathecal administration of the GABA_Aα−1_ agonist, muscimol, wherein the response to noxious colonic distension was inhibited in adult, normosensitive female rats (Sengupta et al., 2013). A positive allosteric modulator of the GABA_B_ receptor significantly inhibited colonic hypersensitivity to distension following systemic, but not intrathecal, administration in female rats (Kannampalli et al., 2017b). The same compound in male mice significantly inhibited acetic acid-induced writhes following oral administration (Kalinichev et al., 2017). The GABA_B_ receptor was also found to be the target for α-conotoxin Vc1.1, which inhibited colonic nociceptive afferent firing as well as the expression of phosphorylated extracellular signal-related kinase, a marker of nociceptive neuronal activation in the dorsal horn of the spinal cord (Castro et al., 2017). Overall, preclinical evidence suggests GABA signaling participates in the maintenance of visceral pain by a loss of inhibitory tone within the spinal cord and/or the central pain matrix. However, lacking in the literature is strong evidence supporting a role of GABA-mediated mechanisms in visceral pain induced by exposure to stress.

### Role of glutamate in stress-induced visceral hypersensitivity

As the major excitatory neurotransmitter, glutamate has both fast ionotropic receptors (AMPA, NMDA, and kainate receptors) and slower metabotropic glutamate (mGlu) receptors that are G-protein coupled (Alexander et al., 2015b,d). Roles for both classes of glutamate receptors in the regulation of acute and chronic pain are well-established (Palazzo et al., 2014; Zhuo, 2017). Another potential therapeutic target to modulate glutamate signaling is the excitatory amino acid transporters (also known as glutamate transporters) that regulate extracellular glutamate concentration (Alexander et al., 2015c). In pre-clinical models, there is a substantial amount of strong data supporting a role for glutamate located at peripheral and central sites in chronic inflammatory- or stress-induced visceral pain. In preclinical models peripheral administration of NMDA antagonists show a dose-dependent reduction in lactic-acid induced writhes (Hillhouse and Negus, 2016). Moreover, in male mice with post-inflammatory colonic hypersensitivity there was an increase in the expression of the NR1 subunit of NMDA receptors in the colon, and the colonic hypersensitivity could be replicated by enema administration of a glutamate agonist and inhibited by an NMDA receptor antagonist (Qi Q. Q. et al., 2016). In another study, supporting a central site of action, zymosan-induced persistent visceral hypersensitivity was associated with an increase in AMPA receptors within the anterior cingulate cortex, with direct infusion of an AMPA receptor antagonist into the anterior cingulate inhibiting the pain responses (Liu S. B. et al., 2015). In another model of colonic hypersensitivity induced by neonatal mustard oil enema, chronic colonic hypersensitivity in rats was associated with increased AMPA and NMDA receptor expression in the anterior cingulate (Zhou et al., 2014). In a model of persistent colonic hypersensitivity induced colonic anaphylaxis, hypersensitivity was demonstrated as increased synaptic facilitation in the anterior cingulate from the thalamus, which was inhibited by local infusion of an antagonist targeting the NR2B subunit of NMDA receptors (Wang et al., 2015). In mice, a prodrug of a mGlu2/3 agonist dose-dependently reduced basal colonic sensitivity, inflammation-induced colonic hypersensitivity, and total writhing behaviors (Johnson et al., 2017). Intrathecal administration of suberoylanilide hydroxamic acid, a histone deacetylase inhibitor, at L6-S2 reversed swim stress-induced colonic hypersensitivity thorough changes in mGlu2/3 receptor expression in female rats (Cao et al., 2016). In a similar fashion, estrogen induced colonic hypersensitivity in ovariectomized female rats was inhibited by intrathecal suberoylanilide hydroxamic acid administration through increases in mGlu2 receptor expression in the spinal cord, providing evidence for epigenetic mechanisms perpetuating chronic phenotypes (Cao et al., 2015). In a rat model of post-infective colonic hypersensitivity, post-infection or acute cold restraint stress increased colonic sensitivity was associated with increased vesicular glutamate transporter 3 expression in the L6-S1 dorsal horn (Yang et al., 2015). Adult male rats with pancreatitis-induced visceral hypersensitivity to mechanical and thermal stimulation had the pain behaviors inhibited by peripheral administration of an mGlu receptor agonist (McIlwrath and Westlund, 2015). Clinical evidence supporting a role for glutamate in stress-induced visceral hypersensitivity is very limited and comes from a few studies in patients with IBS. A recent study found increased colonic mucosal expression of NMDA receptors in IBS patients that was correlated with visceral pain scores (Qi et al., 2017). In another study, an acute oral dose of an NMDA receptor antagonist inhibited temporal summation of second pain in response to noxious thermal stimulation (Zhou et al., 2011). In a follow-up study, a sub-set of IBS patients developed visceral and somatic hypersensitivity induced by repetitive noxious stimulation, which was also inhibited by NMDA receptor antagonism (Verne et al., 2012). IBS patients also demonstrated significant improvement in pain scores following a treatment regimen that included a mixed glutamate receptor uptake enhancer, an NMDA receptor antagonist, an antispasmodic, and a probiotic (Mishra et al., 2014). Although an interesting preliminary investigation, the interpretation of such a clinical study is difficult based upon the limited size of the study and the non-specific nature of this glutamate reuptake enhancer and NMDA receptor antagonist combination.

## Environmental stress in adulthood

Daily life has many stressors, such as finances, illness, or job security, which can be chronic and unpredictable in nature. An individual's response to persistent stressors is, in part, determined by resilience factors, such as social support or underlying genetic polymorphisms (Schilling and Diehl, 2015). However, for vulnerable individuals the inability to adequately cope with chronic stressors can lead to pathological health effects, such as chronic pain syndromes (Gillespie et al., 2009; Leyro et al., 2010). Adults suffering with chronic visceral pain typically have a poor quality of life and require additional healthcare resources to attempt to manage their prolonged visceral pain. Indeed, many sources of chronic pain including stress-induced have been linked to co-morbid psychiatric illnesses such as anxiety and depression, which in turn promote increased pain sensitivity, established a self-reinforcing cycle between chronic stress and chronic pain.

### Animal models to assess stress-induced visceral hypersensitivity in adults

**Figure 2:**
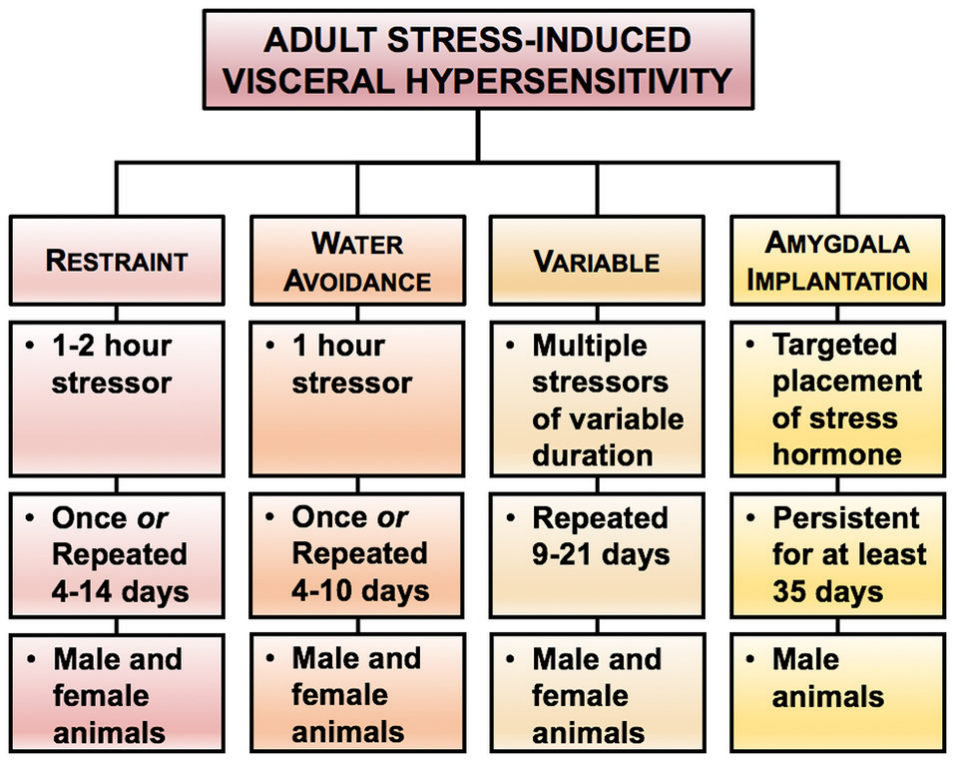
Rodent models of stress-induced visceral hypersensitivity in adult animals. Here we highlight four experimental approaches for increasing visceral sensitivity in adult rodents. In each stress model, we present the duration of the stressor required to produce visceral hypersensitivity and have indicated which sex has been investigated. Please note that the duration and timing of the stressors reflect the range of procedures used within the literature, rather than a specific experimental protocol.

Since the evaluation of spontaneous pain behaviors is difficult in most animal models, pain from the visceral organs is typically evoked by either distension of a hollow organ (esophagus, stomach, colon, uterus, bladder) or by administration of an irritant via intraperitoneal injection or infusion. In response to the stimulus, visceral nociception can be qualitatively evaluated by scoring nocifensive behaviors (abdominal contractions, facial grimace, stretching, writhing). Quantitative measurements of nociceptive stimuli can be conducted through visual counting, or electromyogenic recording, of the visceromotor response or through the use of von Frey filament withdrawal threshold to probing of the abdomen or pubic areas. Limitations of evaluating nocifensive behaviors include that the acute evoked nociceptive responses may not utilize the same signaling mechanisms as chronic pain behaviors; furthermore, without proper acclimatization, the evoked responses can also initiate stress responses that will also influence sensitivity. Evaluation of qualitative measures should be conducted by blinded observers whenever possible to minimize unconscious bias during the behavioral evaluation. While conditioned place preference testing has been developed in models of somatic pain to evaluate spontaneous behaviors and analgesic properties of new therapeutics, the use of this paradigm to evaluate spontaneous visceral pain in animal models has yet to be validated (Navratilova et al., 2013). Multiple models have been developed to evaluate the effect of acute or chronic stressors on visceral sensitivity in adult animals (Figure 2). The key strength of these models is the ability to directly test causal rather than correlative hypotheses through *in vivo* behaviors and *ex vivo* or *in vitro* assays to determine mechanisms responsible for stress-induced chronic visceral pain. The main limitations of these models are that many have low construct validity (rodent stressors do not typically correspond to human stressors), the effect of sex has not necessarily been addressed in each model, and strain differences can affect the results depending on the type and duration of the stressor used to change sensitivity (Dhabhar et al., 1997; Vendruscolo et al., 2004; Girotti et al., 2006). While there are more comprehensive evaluations of animal models for GI disorders, here we focus on discussing the more commonly used models of stress-induced colonic hypersensitivity (Greenwood-Van Meerveld et al., 2015; Johnson and Greenwood-Van Meerveld, 2017). A comparison of the stress models is presented in Table 1. The overall goal of this table is to allow the reader to compare the various stressors and their impact on visceral pain with an emphasis on the strain and sex of the animal and the duration of the stressor. To our knowledge such a comparison between stress models has not been previously reported. However, due to improved standards of data transparency, we were able to attempt this analysis when extrapolating the mean and the variability of the data from either the text and/or the graphical information within the citation was possible. A limitation of this type of effect size comparison across stress models is that we have only used a subset of the published literature based on the ability to determine group sizes, mean and an error estimate from the mean from the published data.

**Table 1:** Comparisons of models to assess stress-induced visceral hypersensitivity in adult animals.

**Species, Strain, Sex**	**Protocol**	**Distension stimulus**	**Hedges' *d* ± se: Sham vs. Stress**	**Reference**
**RESTRAINT STRESS**				
Rat, Sprague Dawley, Male	2 h stress	60 mmHg	3.2 ± 0.8	Ohashi-Doi et al., 2010
	2 h stress/day, 4 days	60 mmHg, 24 h post-stress	4.8 ± 1.0	Shen et al., 2010
Rat, Sprague Dawley, Female	2 h stress	1.2 ml	5.2 ± 1.1	Zhao et al., 2011
Rat, Wistar, Male	2 h stress	1.2 ml	2.1 ± 0.6	Gué et al., 1997
	2 h stress/day, 7 days	60 mmHg	1.9 ± 0.7	Xu et al., 2014
	1 h stress/day, 14 days	60 mmHg, 24 h post-stress	3.4 ± 1.0	Yi et al., 2014
Rat, Wistar, Female	2 h stress	1.2 ml	1.3 ± 0.6	Bradesi et al., 2002
			1.9 ± 0.6	Fioramonti et al., 2003
			1.6 ± 0.6	Ait-Belgnaoui et al., 2005
			1.6 ± 0.5	Agostini et al., 2012
			1.9 ± 0.5	Miquel et al., 2016
		60 mmHg	1.7 ± 0.6	Ait-Belgnaoui et al., 2006
			2.3 ± 0.7	Agostini et al., 2009
			0.6 ± 0.5	Eutamene et al., 2010
			1.2 ± 0.4	Silos-Santiago et al., 2013
			2.5 ± 0.8	Gilet et al., 2014
	2 h stress/day, 4 days	1.2 ml	2.1 ± 0.6	Bradesi et al., 2002
**WATER AVOIDANCE STRESS (WAS)**				
Mouse, C57BL/6J, Male	1 h stress/day, 4 days	0.06 ml	0.8 ± 0.5	Annaházi et al., 2012
			1.2 ± 0.6	Nébot-Vivinus et al., 2014
Rat, Fischer 344, Male	1 h stress	60 mmHg	2.0 ± 0.7	Myers and Greenwood-Van Meerveld, 2012
	1 h stress /day, 7 days	60 mmHg, 24 h post-stress	1.5 ± 0.7	
			2.1 ± 0.7	Tran et al., 2013
			2.1 ± 0.8	Tran et al., 2014
			3.5 ± 1.1	Johnson et al., 2015
Rat, Long Evans, Male	1 h stress	60 mmHg, 24 h post-stress	2.7 ± 0.8	Prusator and Greenwood-Van Meerveld, 2016a
	1 h stress/day, 7 days		2.5 ± 0.7	
Rat, Long Evans, Female	1 h stress		5.2 ± 1.1	
	1 h stress/day, 7 days		5.9 ± 1.3	
Rat, Sprague Dawley, Male	1 h stress	60 mmHg, 24 h post-stress	0.8 ± 0.5	Watson et al., 2012
	1 h stress/day, 10 days		1.6 ± 0.6	Hong et al., 2009
			2.9 ± 0.8	Hong et al., 2011
			2.7 ± 0.8	Hong et al., 2015
			2.5 ± 0.8	Zheng et al., 2015
Rat, Wistar, Male	1 h stress	60 mmHg	1.4 ± 0.6	Nash et al., 2012
		60 mmHg, 24 h post-stress	2.8 ± 0.6	Schwetz et al., 2004
			1.0 ± 0.5	Bradesi et al., 2007
			1.0 ± 0.5	Eutamene et al., 2010
	1 h stress /day, 4 days	60 mmHg	1.8 ± 0.6	Da Silva et al., 2014
	1 h stress/day, 10 days	60 mmHg	0.6 ± 0.3	Bradesi et al., 2005
			4.0 ± 1.1	Wang W. et al., 2017
		60 mmHg, 24 h post-stress	1.2 ± 0.6	Bradesi et al., 2006
			2.8 ± 0.9	Bradesi et al., 2009
			2.0 ± 0.6	Xu et al., 2014
			1.7 ± 0.7	Tang et al., 2015
			5.1 ± 0.6	Sun et al., 2016
Rat, Wistar, Female	1 h stress/day, 10 days	60 mmHg, 24 h post-stress	4.9 ± 1.1	Gilet et al., 2014
**VARIABLE STRESS**				
Mouse, C3H/HeN, Male	19 days	65 mmHg	2.5 ± 0.8	Tramullas et al., 2014
Rat, Sprague Dawley, Male	9 days	60 mmHg	1.5 ± 0.6	Zhou et al., 2012
			1.2 ± 0.6	Chen et al., 2013
			1.9 ± 0.6	Zhang et al., 2014
		60 mmHg, 24 h post-stress	3.6 ± 0.8	Wang et al., 2012
			1.8 ± 0.6	Zhou et al., 2012
			2.7 ± 0.7	Zhang et al., 2014
	21 days	1.2 ml	0.9 ± 0.5	Chen et al., 2009
Rat, Wistar, Male	9 days	60 mmHg	1.3 ± 0.4	Winston et al., 2010
		Area under the curve, 24 h post-stress	2.8 ± 0.7	Winston et al., 2014
Rat, Wistar, Female			3.6 ± 1.0	
**AMYGDALA IMPLANTATION**				
Rat, Fischer 344, Male	7-day post-implant	30 mmHg	1.7 ± 0.6	Greenwood-Van Meerveld et al., 2001
		60 mmHg	2.3 ± 0.7	Myers and Greenwood-Van Meerveld, 2007
			2.6 ± 0.8	Myers and Greenwood-Van Meerveld, 2010a
			2.7 ± 0.8	Myers and Greenwood-Van Meerveld, 2010b
			2.5 ± 0.6	Tran et al., 2012
			2.6 ± 0.8	Johnson and Greenwood-Van Meerveld, 2015
			3.4 ± 0.7	Johnson et al., 2015
	14-day post-implant	60 mmHg	2.4 ± 0.8	Myers and Greenwood-Van Meerveld, 2010b
			2.5 ± 0.6	Tran et al., 2015
	28-day post-implant	60 mmHg	3.0 ± 0.9	Myers and Greenwood-Van Meerveld, 2010b
			2.8 ± 0.7	Johnson et al., 2015

*Due to differences between species, strain, sex, and the methods used to evaluate colonic sensitivity, an effect size for the sham stress vs. stress group was calculated so that the studies could be evaluated and compared on the same scale. For the effect size, Hedges' d with unbiased standard error (se) was estimated from the data presented in the cited paper, based on the formulas 1, 2, 14, and 17 in Nakagawa and Cuthill (2007). Briefly, the magnitude of the effect size allows for a direct comparison between studies from different laboratories. Within each citation, only a single experimental cohort has been reported. Sham and stress exposed animals may have received vehicle treatment(s) before the measurement of colonic sensitivity*.

#### Restraint stress

This model was first demonstrated to induce colonic hypersensitivity to distension following an acute restraint session (Gué et al., 1997). Subsequently, the protocol has been modified by different researchers to restrain the animal either by wrapping the limbs to hinder walking and grooming or by using a tube or cage that prevents turning and grooming for 1–2 h. While the acute session does not model a chronic stressor, this model can be combined with other stressors to evaluate the effects of multiple “hits” on behavior and colonic sensitivity, or the restraint can be repeated each day to induce a more persistent response in strains that do not habituate to the daily stressor (Girotti et al., 2006). Possible modulators of restraint stress-induced colonic hypersensitivity include: cannabinoid type 1 (CB_1_) receptors, CRH receptors, guanylate cyclase-C receptors, neurokinin-1 receptors, neurokinin-3 receptors, nociception/orphanin FQ receptors, protease activated receptors, and serotonin receptors (Gué et al., 1997; Bradesi et al., 2002; Fioramonti et al., 2003; Agostini et al., 2009; Eutamene et al., 2010; Ohashi-Doi et al., 2010; Shen et al., 2010; Zhao et al., 2011; Silos-Santiago et al., 2013; Gilet et al., 2014). Central neural mechanisms influence the nociceptive response to chronic restraint stress as bilateral insular cortex lesions in rats inhibited the stress-induced colonic hypersensitivity (Yi et al., 2014). At a peripheral level, an interaction between colonic hypersensitivity and colonic permeability was demonstrated by reversal of both stress-induced changes following administration of a tight-junction inhibitor or myosin light chain kinase inhibitor, but not an endothelial cell adhesion molecule inhibitor (Ait-Belgnaoui et al., 2005; Winchester et al., 2009). There is also evidence suggesting a potential role for the microbiome in the development of stress-induced colonic hypersensitivity. However, to date there have been only a limited number of studies investigating the direct interactions of the gut microbiota and its metabolites on restraint stress induced pain and nociceptive processes. There is preclinical data showing that antibiotic treatment or administration of specific probiotic genera such as *Lactobacillus* or *Bifidobacterium* inhibit partial restraint stress-induced visceral hypersensitivity (Ait-Belgnaoui et al., 2006; Agostini et al., 2012; Xu et al., 2014; Miquel et al., 2016; Darbaky et al., 2017). However, the ability to translate positive preclinical findings with probiotics into effective therapeutics has proven difficult due in part to the huge diversity in the microbiome between rodents and patients.

#### Water avoidance stress (WAS)

WAS has been used to model both acute and chronic effects of a psychological stressor on colonic sensitivity. The typical procedure is to place the rodent on a platform surrounded by water in an unescapable enclosure for 1 h per day, either acutely or for 7–10 days (Bradesi et al., 2005; Hong et al., 2009; Tran et al., 2013). Colonic sensitivity to distension is then typically evaluated immediately or 24-h post the final WAS procedure (Eutamene et al., 2010; Myers and Greenwood-Van Meerveld, 2012; Nash et al., 2012; Watson et al., 2012; Tran et al., 2014; Prusator and Greenwood-Van Meerveld, 2016a). Possible modulators of WAS-induced colonic hypersensitivity include: CRH receptors, dopamine-2 receptors, guanylate cyclase-C receptors, potassium chloride co-transporter, protease activated receptor-4, neurokinin-1 receptors, serotonin receptors, transient receptor potential cation channel subfamily V member 1 (TRPV1) receptors, and vasopressin-3 receptor (Schwetz et al., 2004; Bradesi et al., 2006, 2007, 2009; Eutamene et al., 2010; Annaházi et al., 2012; Nash et al., 2012; Gilet et al., 2014; Tang et al., 2015; Sun et al., 2016). Within limbic brain circuits, glucocorticoid receptor (GR), mineralocorticoid receptor (MR) and CRH mediate WAS-induced colonic hypersensitivity (Myers and Greenwood-Van Meerveld, 2012; Tran et al., 2013, 2014; Johnson et al., 2015). In the dorsal root ganglia that innervate the distal colon, epigenetic mechanisms differentially affect expression of GR, CB_1_, and TRPV1 to induce colonic hypersensitivity following WAS (Hong et al., 2009, 2011, 2015). Through the use of antibiotics, prebiotics, probiotics, and anti-fungal agents, there is preclinical evidence supporting a role for microbiota to inhibit WAS-induced colonic hypersensitivity (Da Silva et al., 2014; Nébot-Vivinus et al., 2014; Xu et al., 2014; Botschuijver et al., 2017; Wang W. et al., 2017). Recently, WAS has been used as a model of stress-induced bladder pain to investigate mechanisms of visceral pain associated with interstitial cystitis or bladder pain syndrome (Lee et al., 2015; Ackerman et al., 2016; Matos et al., 2017; Wang Z. et al., 2017).

#### Variable stress

Both restraint and water avoidance are homotypic stressors, and some rodent strains will adapt to repeated stress exposure. To prevent adaptation, rodent models termed, heterotypic intermittent stress or heterotypic chronic stress (HeCS), have been developed in which the animals are exposed to variable stressors (cold restraint, water avoidance, or forced swim) presented randomly over multiple days to induce persistent colonic hypersensitivity. While used less frequently, these stress paradigms have demonstrated roles for β_2_ adrenergic receptors, brain-derived neurotrophic factor, cystathionine β-synthetase, endorphins, and nerve growth factor in the stress-induced colonic hypersensitivity (Winston et al., 2010, 2014; Wang et al., 2012; Zhou et al., 2012; Chen et al., 2013; Zhang et al., 2014). Additional models of chronic variable stress implicated mast cell mediators and toll-like receptor 4 signaling as factors induced by stress to promote chronic colonic hypersensitivity (Chen et al., 2009; Tramullas et al., 2014).

#### Amygdala implantation

Manipulation of limbic brain circuitry that integrates stress and pain processing is sufficient to change colonic sensitivity in rats. In our team, we have stereotaxically targeted the CeA with CORT micropellets to induce colonic hypersensitivity to distension and anxiety-like behavior (Greenwood-Van Meerveld et al., 2001). After 7 days of exposure of the CeA to the CORT micropellet, there is also increased blood-oxygen utilization in response to colonic distension throughout the brain, which is similar to heightened brain activation induced by colonic distension in IBS patients (Naliboff et al., 2003; Wilder-Smith et al., 2004; Johnson et al., 2010). Mechanistically, there are non-redundant roles for both GR and MR signaling and CRH type 1 (CRH_1_) receptors mediating the persistent colonic hypersensitivity (Myers and Greenwood-Van Meerveld, 2007, 2010a,b; Johnson et al., 2012; Tran et al., 2012). Furthermore, directly manipulating GR, MR, or CRH expression within the CeA had a profound effect on colonic sensitivity illustrating the importance of the CeA in visceral pain processing (Johnson and Greenwood-Van Meerveld, 2015; Johnson et al., 2015). Chronic changes in GR and CRH expression following the CORT micropellet implantation on the CeA were induced by an epigenetic mechanism involving deacetylation of the GR promoter causing increases in CRH expression, leading to the chronic colonic hypersensitivity (Tran and Greenwood-Van Meerveld, 2012; Tran et al., 2015). Thus, central dysfunction of limbic circuity induces colonic hypersensitivity without manipulation of the colon.

## Relationship between early life stress (ELS) and chronic visceral pain in later life

Early childhood is a pivotal period for the development of the specific brain circuitry regulating stress and nociception. In the United States, at least 1 in 10 children will experience some form of physical and/or psychological abuse that will bias the development of their pain neurocircuitry toward enhanced pain perception in adulthood (Anda et al., 2006; Bradford et al., 2012). The “multiple hit” model has gained popularity to explain how the complex interaction between genetic and epigenetic risk factors and adverse childhood experiences (early life stresses) induce adult pathologies such as mood disorders or the development of chronic pain. With the first “hit” being *in utero* development or genetic predisposition, ELS before puberty acts as a second “hit” to cause maladaptation of the stress axis to stressors that can predispose the individual to heightened pain perception. During puberty (potentially a third “hit”), the surge of hormones, especially estrogen and progesterone, further sensitizes stress and pain circuitry promoting the development of functional visceral pain disorders such as IBS in adulthood (Meleine and Matricon, 2014). Each “hit,” such as abuse, parental care, poor diet, psychological disorders, or social stressors, drives the stress and pain circuitry toward persistent sensitization leading to IBS symptomology due to a dysregulation of the brain-gut axis (Miranda and Saps, 2014).

### Animal models to assess ELS-induced visceral hypersensitivity

**Figure 3:**
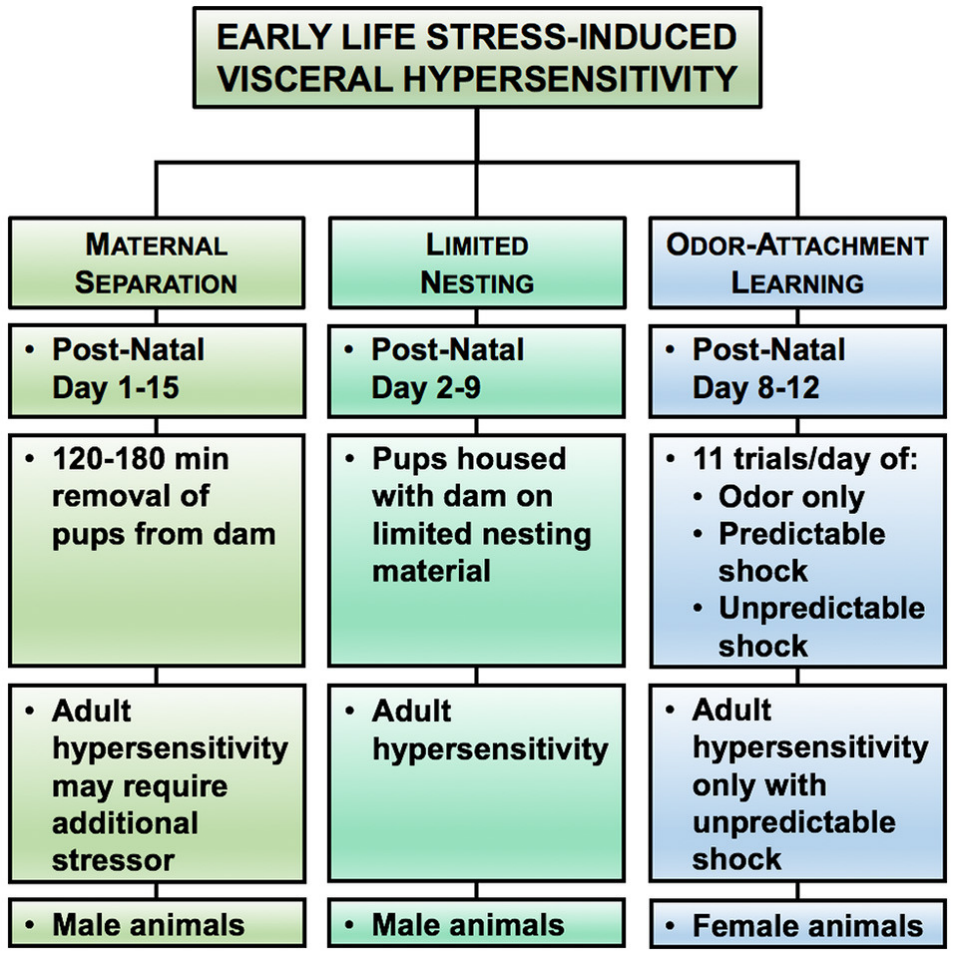
Rodent models of early life stress (ELS)-induced visceral hypersensitivity. Here we highlight three experimental approaches for increasing visceral sensitivity in adult rodents in response to early life stress. In each model, we have summarized the typical post-natal period of the stress exposure, the duration of the stressor, and the effect on colonic sensitivity in adulthood, along with the sex of the rat reliably showing colonic hypersensitivity. Please note that the duration and timing of the ELS reflects the range of procedures used within the literature, rather than a specific experimental protocol.

Animal models have provided evidence that pain in early life is capable of priming nerves to be more excitable in response to nociceptive stimuli in adulthood (Beggs et al., 2012). Due to the variety of ELS models, the mechanism underlying the development of chronic visceral pain in adulthood will be influenced by the type, duration, and developmental timing of the initial insult. While most of the ELS models induce chronic visceral hypersensitivity, the nature of the ELS (modeling neglect, poverty, or abuse) produces chronic, sexually dimorphic changes in behaviors that can be exploited to model different life experiences in adulthood in patients with chronic abdominal pain (Figure 3) (Prusator and Greenwood-Van Meerveld, 2016b). A comparison of the ELS models discussed below is presented in Table 2 with the same caveats as previously described for Table 1.

**Table 2:** Comparisons of models to assess early life stress (ELS)-induced visceral hypersensitivity in adult animals.

**Species, Strain, Sex**	**Protocol**	**Distension stimulus**	**Hedges' *d* ± se: Sham vs. Stress**	**Reference**
**MATERNAL SEPARATION (MS)**				
Mouse, C57BL/AJ, Male	180 min separation, PN 2-14	0.1 ml	1.1 ± 0.5	Miquel et al., 2016
Mouse, C57BL/10JNju, Both	180 min separation x 2/day, PN 2-15	40 mmHg	1.9 ± 0.6	Tang et al., 2017
Rat, Long Evans, Male	180 min separation, PN 2-14	60 mmHg	0.5 ± 0.3	Coutinho et al., 2002
			1.0 ± 0.4	Prusator and Greenwood-Van Meerveld, 2016b
	180 min separation, PN 2-14, sensitivity testing after 1 h WAS	60 mmHg	0.7 ± 0.4	Coutinho et al., 2002
		2.0 ml	1.6 ± 0.6	van den Wijngaard et al., 2009
		Area under the curve	2.5 ± 0.6	van den Wijngaard et al., 2012
			2.6 ± 0.7	Stanisor et al., 2013
			4.8 ± 1.0	Botschuijver et al., 2017
Rat, Wistar, Male	120 min separation, PN 1-14	1.2 ml	1.4 ± 0.5	Rosztóczy et al., 2003
Rat, Wistar, Female			1.3 ± 0.5	
**LIMITED NESTING**				
Rat, Long Evans, Male	PN 2-9	60 mmHg	2.5 ± 0.6	Prusator and Greenwood-Van Meerveld, 2016b
Rat, Sprague Dawley, Male	PN 2-9	60 mmHg	1.4 ± 0.5	Prusator and Greenwood-Van Meerveld, 2015
Rat, Wistar, Male	PN 2-9	60 mmHg	1.1 ± 0.5	Holschneider et al., 2016
Rat, Wistar, Female			1.1 ± 0.5	
**ODOR-ATTACHMENT LEARNING**				
Rat, Long Evans, Both	PN 8-12	60 mmHg	1.5 ± 0.7	Tyler et al., 2007
Rat, Long Evans, Female			1.4 ± 0.6	Chaloner and Greenwood-Van Meerveld, 2013
			5.2 ± 1.2	Prusator and Greenwood-Van Meerveld, 2016a
			1.3 ± 0.5	Prusator and Greenwood-Van Meerveld, 2016b
			5.2 ± 1.3	Prusator and Greenwood-Van Meerveld, 2017

*Due to differences between species, strain, sex, and methods used to evaluate colonic sensitivity, an effect size for the sham stress vs. stress group was calculated so that the studies could be evaluated and compared on the same scale. All colonic sensitivity assessments were performed in adult animals. For the effect size, Hedges' d with unbiased standard error (se) was estimated from the data presented in the cited paper, based on the formulas 1, 2, 14, and 17 in Nakagawa and Cuthill (2007). Briefly, the magnitude of the effect size allows for a direct comparison between studies from different laboratories. Within each citation, only a single experimental cohort has been reported. Sham and stress exposed animals may have received vehicle treatment(s) before the measurement of colonic sensitivity. PN, post-natal day*.

#### Maternal separation (MS)

Adult male rats exposed to MS as neonates develop colonic hypersensitivity to distension or display colonic hypersensitivity following an adult stressor (second “hit”) (Coutinho et al., 2002; Rosztóczy et al., 2003; van den Wijngaard et al., 2009, 2012; Stanisor et al., 2013). MS models neglect through the prolonged separation of the dam from the pups. Upon return of the pups, the dam's behavior is altered such that the quality of care is reduced, which primes aberrant responses of the HPA axis.

#### Limited nesting

In an attempt to model impoverished care in early life, rodents are exposed to a nest with reduced bedding material (Drake and Pandey, 1996). Without adequate resources to build a nest for her pups, the quality of the dam's care for the pups is abnormal causing abnormal activation of the HPS axis that persists into adulthood (Gilles et al., 1996; Avishai-Eliner et al., 2001). Following exposure to limited nesting as neonates, colonic hypersensitivity is predominantly seen in adult males (Prusator and Greenwood-Van Meerveld, 2015, 2016b; Holschneider et al., 2016). There is also evidence for altered connectivity and function (CRH and GR expression) of limbic and pain circuits in adult rats exposed to limited nesting (Avishai-Eliner et al., 2001; Ivy et al., 2008; Rice et al., 2008; Holschneider et al., 2016).

#### Odor-attachment learning

Using a classical conditioning paradigm, the odor-attachment learning paradigm attempts to model attachment to an abusive caregiver by exposing the neonatal pups to predictable or unpredictable odor-shock pairings (Sullivan et al., 2000; Tyler et al., 2007; Sevelinges et al., 2011). The odor-attachment learning model exploits the stress hyporesponsive period in the neonatal pups (post-natal day 8–12) to induce an attachment to the conditioning odor in the predictable shock group without causing the pups to form an association or an aversion to conditioning odor in the unpredictable shock group (Camp and Rudy, 1988; Moriceau and Sullivan, 2004; Moriceau et al., 2006). Only female rats exposed to unpredictable shock develop an estrogen-dependent colonic hypersensitivity, whereas colonic sensitivity in the female rats in the predictable shock and odor only control groups and in all male pups regardless of conditioning exposure resemble normosensitive rats (Chaloner and Greenwood-Van Meerveld, 2013; Prusator and Greenwood-Van Meerveld, 2016b). In addition to systemic estrogen, colonic hypersensitivity in the female rats exposed to unpredictable shock is mediated by GR and CRH activation of CRH_1_ within the CeA (Prusator and Greenwood-Van Meerveld, 2017). Interestingly, female rats in both the predictable and unpredictable shock groups demonstrate a similar hypersensitive response to colonic distension after a chronic stressor, suggesting that the chronic stress induces a phenotypic switch from resilient to vulnerable in the predictable shock group (Prusator and Greenwood-Van Meerveld, 2016a).

## Sex-linked differences in stress-induced visceral pain sensitivity

Within the United States, female patients receive a diagnosis of IBS and other functional pain disorders at twice the rate of males (Chial and Camilleri, 2002; Chang et al., 2006; Heitkemper and Jarrett, 2008). One factor that may explain the increased incidence in females is that gastrointestinal symptoms, such changes in bowel habits, bloating and abdominal pain, are affected by hormone fluctuations during the menstrual cycle (Laessle et al., 1990; Whitehead et al., 1990; Kane et al., 1998). While clinical studies support a role for sex hormones interacting with IBS symptomology, mechanistic studies that provide evidence for specific signaling pathways mediating visceral pain in females are lacking (Ouyang and Wrzos, 2006; Heitkemper and Chang, 2009). A history of early life adversity is an additional factor that may contribute to the increased diagnosis of IBS in females (Drossman et al., 1990; Talley et al., 1994; Bradford et al., 2012). In response to colorectal distension, female IBS patients typically report lower thresholds for pain/discomfort or more pain at similar distension pressures compared to their male counterparts with IBS (Adeyemo et al., 2010; Meleine and Matricon, 2014). Functional imaging studies have been conducted in an attempt to identify sex differences between healthy volunteers and IBS patients. A summary of imaging studies in response to colorectal distension found consistent abnormalities in activation of the amygdala, insula, cingulate, and prefrontal cortex between IBS patients and healthy volunteers (Weaver et al., 2016). Similarly, in IBS patients, females demonstrated altered amygdala and cingulate activation compared to males (Naliboff et al., 2003). A newer modality in brain imaging is the analysis of resting state functional connectivity which aims to identify default networks influencing behaviors and pain perception. In studies comparing resting state functional connectivity in IBS patients to healthy controls, significant alterations in amygdala connections between the insula and other cortical regions, altered cingulate-cortical connections, and alterations in amygdala-insula and cingulate-thalamic connections were found to be specific to females with IBS and visceral hypersensitivity (Ma et al., 2015; Qi, R. et al., 2016; Weng et al., 2016; Icenhour et al., 2017). Additionally, a history of ELS influences resting state functional connectivity in both male and female IBS patients (Gupta et al., 2014). Overall, these imaging studies verify that the central pain matrix is differentially activated in females and males with regard to visceral sensation, in part due to the influence of sex hormones on neuronal sensitivity (Chang et al., 2006; Voß et al., 2013). Animal data support these clinical observations. Specifically, female animals display increased colonic sensitivity following stress exposure in comparison to male animals (Kayser et al., 1996; Chaloner and Greenwood-Van Meerveld, 2013). Furthermore, the phase of the estrus cycle can affect colonic sensitivity in female rodents, with hypersensitivity during proestrus/estrus phases with high circulating estrogen and progesterone vs. diestrus/metestrus with the lowest circulating hormone levels (Sapsed-Byrne et al., 1996; Ji et al., 2008). The ability to recapitulate clinical observations in experimental models will provide a foundation for future studies investigating the basic mechanisms underlying stress-induced visceral hypersensitivity.

In female rats the central mechanisms modulating visceral hypersensitivity have been investigated in experimental models. Elevating amygdala CORT with stereotaxically placed micropellets on the CeA, we have observed colonic hypersensitivity during diestrus or following ovariectomy, but not during proestrus (Gustafsson and Greenwood-Van Meerveld, 2011). Furthermore, in ovariectomized female or male rats, stereotaxic implantation onto the CeA of micropellets containing estrogen or progesterone induced colonic hypersensitivity (Gustafsson and Greenwood-Van Meerveld, 2011; Myers et al., 2011). Aside from its reproductive role, estrogen is a key mediator of brain development and plays a role in plasticity in nociceptive circuits (Handa et al., 1994; Fitch and Denenberg, 1998). For example, within the medial amygdala, estrogen causes μ-opioid receptor internalization and within nociceptive circuitry estrogen can compete with GR to affect CRH signaling to promote increased sensitivity to peripheral sensations (Vamvakopoulos and Chrousos, 1993; Uht et al., 1997; Eckersell et al., 1998; Miller et al., 2004). At the level the dorsal horn of the spinal cord, estrogen can modify expression and function of NMDA receptors and mGlu2 receptors to effect ascending visceral pain signaling (Tang et al., 2008; Cao et al., 2015; Ji et al., 2015). In females, estrogen-induced changes in glutamate receptor function can promote the sensitization of the nociceptive circuits to induce or exacerbate colonic hypersensitivity. These same mechanisms amplifying colonic hyperalgesia can be invoked in male rodents through the exogenous administration of estrogen (Aloisi and Ceccarelli, 2000; Aloisi and Bonifazi, 2006). In summary, while the interactions between sex hormones and pain circuitry is complex, steroid hormones can promote the development of chronic hypersensitivity and likely contribute to the sexual dimorphism observed in patients with functional pain disorders such as IBS. Moreover, while sex hormones can contribute to enhanced pain signaling, other factors such as individual differences in stress exposure and coping behaviors, socioeconomic factors, and genetic predisposition contribute to the development of chronic visceral pain (Heitkemper and Jarrett, 2008; Meleine and Matricon, 2014).

## Epigenetic mechanisms mediating stress-induced chronic visceral pain

**Figure 4:**
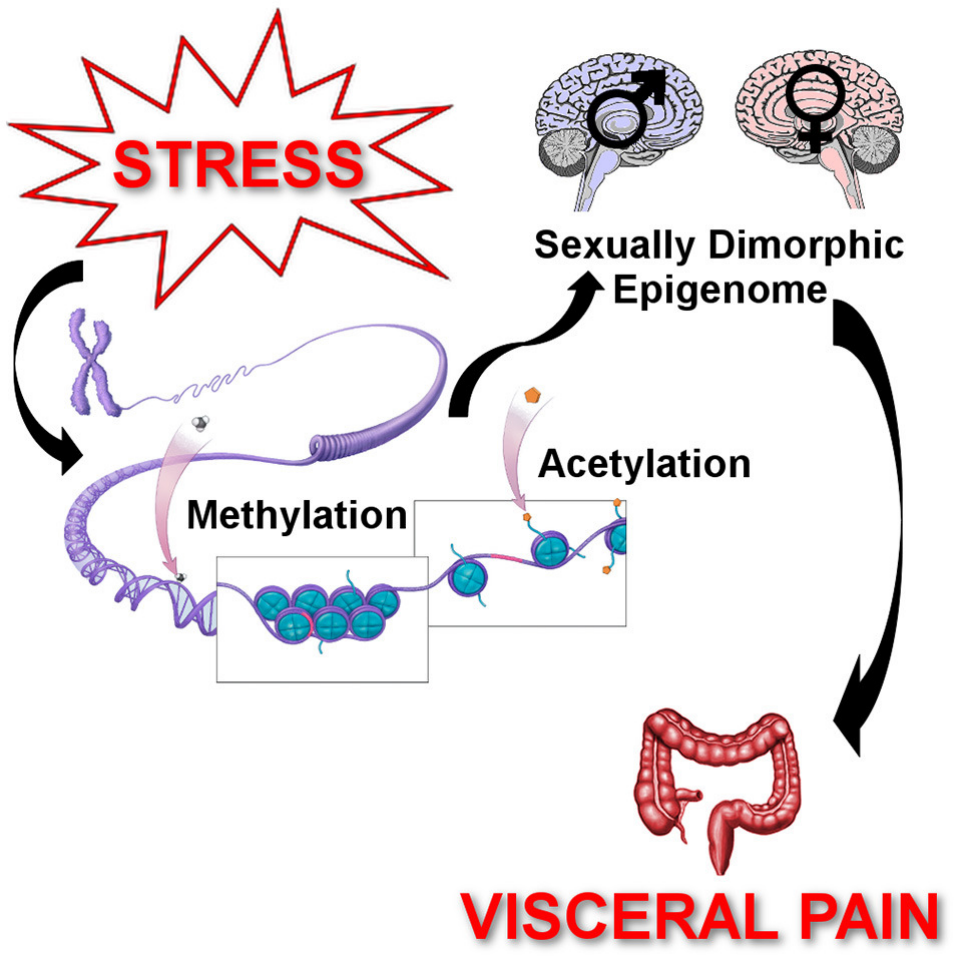
Epigenetic regulation of chronic stress-induced visceral pain. Epigenetics describes the processes by which the environment influences gene expression to cause persistent changes in behaviors. Stressors (early life stress, adult stressors, or both) induce changes in the methylation status of DNA promoter regions to enhance or repress transcription rates. Stressors also change the state of histone acetylation around the gene promoter regions to facilitate or hinder the binding of the transcription complex, which also affects gene transcription. These stress-induced changes in DNA methylation and histone acetylation cause changes in gene expression that persist well beyond the duration of the stressor. Additionally, due to hormonal differences across the lifespan, sex differences in response to stressors can also modify the epigenetically induced changes in gene expression. The net effect is the development of chronic visceral pain following stressors that persist through the individual's lifetime due to epigenetically induced changes in gene expression leading to enhanced neuronal sensitivity.

While polymorphisms or random mutations within a genotype can bias an individual toward pathophysiology, the contribution of the environment will ultimately determine the resilience or vulnerability to stress-induced visceral pain by affecting how genes are expressed. Following the resolution of an injury, many patients suffer from chronic visceral pain suggesting that epigenetic mechanisms may play a role in the persistent nature of the pain. Epigenetics describes variations in phenotype expression due to environmental influences in the absence of mutations within the genomic DNA (Waddington, 1942). While modifications to chromatin, such as acetylation or methylation of histones, are the most studied epigenetic mechanisms, modification of the DNA bases leading to enhanced or repressed transcription also influence overall gene expression (Bernstein et al., 2007). The definition of epigenetics has been expanded to include regulatory RNA sequences (microRNA [miRNA] or small non-coding RNA) due to their ability to affect mRNA translation (Farh et al., 2005; Zhang and Banerjee, 2015). While epigenetic mechanisms have been identified in some chronic neuropathic or inflammatory pain disorders, epigenetic regulation of chronic visceral pain is still an emerging field of research interest (Greenwood-Van Meerveld et al., 2016; Ligon et al., 2016). Histone acetylation and DNA methylation are the other epigenetic mechanisms that can induce persistent changes in gene expression throughout pain circuitry to promote chronic visceral pain (Figure 4). In the first report of an epigenetic regulation of stress-induced visceral pain, we found in male rats that following repeated exposure to a water avoidance stress paradigm there were multiple epigenetic changes within the CeA including an increased methylation of the glucocorticoid receptor (GR) promoter region and decreased methylation of the CRH promoter region, combined with a decrease in GR mRNA expression and an increase in CRH mRNA expression (Tran et al., 2013). Concurrent with the stress-induced changes in DNA methylation, daily intracerebroventricular administration of the histone deacetylase inhibitor trichostatin A during the stress exposure inhibited the stress-induced colonic hypersensitivity (Tran et al., 2013). These findings provide evidence that repeated psychological stressors induce changes in brain circuits that integrate stress and pain signaling through epigenetic mechanisms. Building upon these initial findings, we found that colonic hypersensitivity induced by elevated amygdala corticosterone (CORT) was associated with persistent decreases in GR expression and persistent increases in CRH expression within the CeA (Tran and Greenwood-Van Meerveld, 2012). In our subsequent investigation of the epigenetic mechanisms responsible for the persistent colonic hypersensitivity and changes in gene expression, we used chromatin immunoprecipitation assays to show that the reduction in GR expression in the CeA was due a decrease in acetylation of histone-3 at lysine-9 (ac-H3K9) at the GR promoter, leading to a reduction of GR expression (Tran et al., 2015). With this loss of GR there was a reduction in its binding to a negative response element in the CRH promoter, permitting an increase in AP-1 binding to a positive regulatory element thereby increasing CRH expression within the CeA (Tran et al., 2015). Furthermore, the loss of ac-H3K9 could be due to increase activity of the histone deacetylase sirtuin-6 that was recruited to the GR promoter region by CORT-induced nuclear factor kappa B signaling (Tran et al., 2015). Intra-CeA infusions of trichostatin A or suberoylanilide hydroxamic acid inhibited the CORT-induced colonic hypersensitivity by restoring ac-H3K9 at the GR promoter to prevent the decrease in GR expression (Tran et al., 2015). In another study, Hong and coworkers investigated whether peripheral epigenetic mechanisms play any role in stress-induced visceral hypersensitivity. Following exposure to the water avoidance stress paradigm, analysis of isolated L6-S2 dorsal root ganglia (DRG) revealed increased methylation of the GR promoter region due to increased expression of DNA methyltransferase 1. Together these changes led to a decrease in GR expression and a downregulation of cannabinoid type 1 receptor (CB_1_), which has positive glucocorticoid response elements upstream of the transcription start site, with specific binding verified by chromatin immunoprecipitation assay (Hong et al., 2015). Additionally, there was a specific increase in acetylation of histone 3 around the TRPV1 promoter, due to increased expression of the histone acetyltransferase EP300, leading to increased expression of TRPV1 within the L6-S2 DRG (Hong et al., 2015). Building upon these findings, the same team showed that systemic administration of a GR antagonist or intrathecal administration of siRNA targeting DNA methyltransferase 1, EP300, or TRPV1 attenuated WAS-induced colonic hypersensitivity. Conversely, siRNA targeting CB_1_ was found to induce colonic hypersensitivity in non-stressed rats (Hong et al., 2015). Further support for epigenetic mechanisms in stress-induced visceral hypersensitivity in female rats showed that intrathecal suberoylanilide hydroxamic acid administration inhibited stress-induced colonic hypersensitivity through increases in expression of the mGlu2 receptor within the lumbosacral spinal cord due to specific increases in ac-H3K9 upstream of the transcriptional start site for the mGlu2 receptor (Cao et al., 2015, 2016).

Adverse early environmental experiences, such as abuse, neglect, or sexual trauma, are risk factors for the development of chronic visceral pain disorders through epigenetic remodeling of pain pathways (Bradford et al., 2012; Liu et al., 2017). Although the epigenetic mechanism was not identified, maternal separation (MS)-induced susceptibility to stress-induced colonic hypersensitivity could be transmitted to the F2 generation (van den Wijngaard et al., 2013). In a similar model of MS, peripheral administration of suberoylanilide hydroxamic acid inhibited the MS-induced increase in pain behaviors to distension and increased acetylation of histone-4 at lysine-12 in the L5-S2 spinal cord (Moloney et al., 2015). In a two “hit” model of post-inflammatory early life stress, colonic hypersensitivity was induced by neonatal and adult colonic inflammation, which caused an increase in brain-derived neurotrophic factor (BDNF) expression in the lumbosacral spinal cord due to increases in ac-H3K9 and acetylation of histone-4 at lysine-12 (Aguirre et al., 2017). In another important preclinical study performed by Winston and colleagues, rat dams were exposed to HeCS for the final 10 gestational days and then the HeCS protocol was repeated in the adult offspring. Both male and female offspring of HeCS dams were hypersensitive to distension in adulthood, and while both sexes showed an exacerbation of colonic hypersensitivity immediately following the HeCS protocol, only female offspring developed a persistent colonic hypersensitivity (Winston et al., 2014). Furthermore, in female rats exposed to HeCS from dams that underwent HeCS, there was increased expression of BDNF due to increased ac-H3K9 of the BDNF promoter. These changes in expression and colonic hypersensitivity were inhibited by intrathecal dosing of histone acetyltransferase inhibitors (Winston et al., 2014).

Clinical samples from patients with visceral pain have focused on miRNA targets due to their potential to serve as clinical biomarkers. Whole blood samples from IBS patients and controls identified miR-150 and miR-342-3p as differentially expressed with the potential to affect pain signaling pathways (Fourie et al., 2014). In another study, biopsies from diarrhea-predominate IBS patients found that miR-29 expression positively correlated with increased colonic permeability. Although visceral sensitivity was not evaluated, increased permeability could potentially promote peripheral and central sensitization leading to increased visceral hypersensitivity (Zhou et al., 2010, 2015; Zhou and Verne, 2011; Camilleri et al., 2012). Colonic biopsy samples from IBS patients were also found to have increased expression of miR-24 (Liao et al., 2016), however its role in stress-induced visceral pain remains to be studied. As a negative regulator of transient receptor potential cation channel subfamily V member 1 (TRPV1) expression, miR-199 expression in colonic biopsies was negatively correlated with visceral pain scores and TRPV1 expression in diarrhea-predominate IBS patients (Zhou Q. et al., 2016). Preclinical studies in rat models examined miRNA expression in the spinal cord and identified miR-17-5p as a possible mediator of water avoidance stress-induced colonic hypersensitivity (Sengupta et al., 2013; Bradesi et al., 2015; Zhang et al., 2017). In summary, life experiences along with a person's genetic make-up determines their vulnerability or resilience to developing chronic stress-induced pain disorders such as IBS. The interactions between genes and environments, termed epigenetics, influence long-term stress reactivity and pain sensitivity which can lead to the development of pathophysiology with each “hit” received by an individual.

## Summary and conclusion

Patients suffering from chronic visceral pain experience a significant reduction in their quality of life, utilize more healthcare resources, and have few therapeutic options. Visceral pain can be initiated from the “bottom-up” by a disturbance within the periphery such as an infection or injury, or could be initiated from the “top-down” by a pathophysiologic response to severe or repeated stressors. Indeed, there is significant overlap of neural circuity that process sensations of pain or stress, such as the amygdala, the insula, or areas of the cingulate, along with common neurotransmitters and their receptors, such as GR or CRH, that are expressed within the GI tract on resident immune cells or intrinsic nerves and within dorsal root ganglia, the spinal cord, and throughout the brain. The major neuroendocrine stress hormone, CORT, which is secreted in response to activation of the HPA axis by stressors, acts at GR and MR receptors throughout the body, but can also promote enhanced sensitivity of neurons to both noxious and innocuous stimuli, which in turn promotes the development of chronic pain. In addition to their chronic pain disorder, patients may have reduced coping skills to typical life stressors. To investigate the mechanisms and identify new therapeutics for chronic visceral pain, multiple animal models of stress-induced colonic hypersensitivity have been developed. While each model employs a different adult stressor (physical, psychological, or both), applied for different durations and/or repetitions, these various experimental approaches induce consistent visceral hypersensitivity in rodent models. An additional and important risk factor for the development of chronic visceral pain is exposure to early life adverse environments, such as abuse, neglect, or poverty. These early life stressors are thought to prime the developing stress and pain circuity within the nervous system to become sensitized in response to stimuli. Rodent models have been used to model specific aspects of early life stress to identify vulnerability and resilience factors depending on whether the adult animal develops chronic colonic hypersensitivity. Sex is also a significant factor in the development of chronic stress-induced pain. Females are twice as likely to be diagnosed with a chronic visceral pain disorder, and are more likely to have a history of early life stress. Moreover, since sex hormones can modulate neuronal sensitivity and synaptic connections, females may have a biological bias toward chronic visceral pain. Finally, epigenetics are the tools the body uses to imprint positive and negative environmental experiences onto the genotype of an individual to produce persistent phenotypes. The overall resilience or vulnerability of an individual is the net effect of epigenetic processes that can promote or dampen pain sensitivity, stress reactivity, and coping with adversity to either result in a healthy individual or one that suffers from chronic visceral pain in response to a lifetime of stressors.

## Author contributions

Both authors made a substantial, direct and intellectual contribution to the work, and approved it for publication.

### Conflict of interest statement

The authors declare that the research was conducted in the absence of any commercial or financial relationships that could be construed as a potential conflict of interest.

## Abbreviations

IBS: irritable bowel syndrome
ELS: early life stress
GI: gastrointestinal
DRG: dorsal root ganglia
AMPA: α-amino-3-hydroxy-5-methyl-4-isoxazolepropionic acid
NMDA: N-methyl-d-aspartate
HPA: hypothalamic-pituitary-adrenal
CORT: corticosterone or cortisol
CRH: corticotropin-releasing hormone
MR: mineralocorticoid receptor
GR: glucocorticoid receptor
CeA: central nucleus of the amygdala
WAS: water avoidance stress
CB_1_: cannabinoid receptor type 1
TRPV1: transient receptor potential cation channel subfamily V member 1
CRH_1_: CRH type 1 receptor
CRH_2_: CRH type 2 receptor
2-AG: 2-arachidonoylglycerol
CB_2_: cannabinoid receptor type 2
FAAH: fatty acid amide hydrolase
MAGL: monoacylglycerol lipase
siRNA: small interfering RNA
GABA: gamma-amino butyric acid
GABA_A_: GABA type A receptor
GABA_B_: GABA type B receptor
mGlu: metabotropic glutamate receptor
HeCS: heterotypic chronic stress
BDNF: brain-derived neurotrophic factor
MS: maternal separation
miRNA: microRNA
ac-H3K9: acetylation of histone-3 at lysine-9.
